# Electroporation-Induced Electrosensitization

**DOI:** 10.1371/journal.pone.0017100

**Published:** 2011-02-09

**Authors:** Olga N. Pakhomova, Betsy W. Gregory, Vera A. Khorokhorina, Angela M. Bowman, Shu Xiao, Andrei G. Pakhomov

**Affiliations:** 1 Frank Reidy Research Center for Bioelectrics, Old Dominion University, Norfolk, Virginia, United States of America; 2 Department of Electrical and Computer Engineering, Old Dominion University, Norfolk, Virginia, United States of America; University of Queensland, Australia

## Abstract

**Background:**

Electroporation is a method of disrupting the integrity of cell membrane by electric pulses (EPs). Electrical modeling is widely employed to explain and study electroporation, but even most advanced models show limited predictive power. No studies have accounted for the biological consequences of electroporation as a factor that alters the cell's susceptibility to forthcoming EPs.

**Methodology/Principal Findings:**

We focused first on the role of EP rate for membrane permeabilization and lethal effects in mammalian cells. The rate was varied from 0.001 to 2,000 Hz while keeping other parameters constant (2 to 3,750 pulses of 60-ns to 9-µs duration, 1.8 to 13.3 kV/cm). The efficiency of all EP treatments was minimal at high rates and started to increase gradually when the rate decreased below a certain value. Although this value ranged widely (0.1–500 Hz), it always corresponded to the overall treatment duration near 10 s. We further found that longer exposures were more efficient irrespective of the EP rate, and that splitting a high-rate EP train in two fractions with 1–5 min delay enhanced the effects severalfold.

**Conclusions/Significance:**

For varied experimental conditions, EPs triggered a delayed and gradual sensitization to EPs. When a portion of a multi-pulse exposure was delivered to already sensitized cells, the overall effect markedly increased. Because of the sensitization, the lethality in EP-treated cells could be increased from 0 to 90% simply by increasing the exposure duration, or the exposure dose could be reduced twofold without reducing the effect. Many applications of electroporation can benefit from accounting for sensitization, by organizing the exposure either to maximize sensitization (e.g., for sterilization) or, for other applications, to completely or partially avoid it. In particular, harmful side effects of electroporation-based therapies (electrochemotherapy, gene therapies, tumor ablation) include convulsions, pain, heart fibrillation, and thermal damage. Sensitization can potentially be employed to reduce these side effects while preserving or increasing therapeutic efficiency.

## Introduction

Electroporation of cell membranes by EPs, also known as electropermeabilization, has been extensively studied for several decades. Experimental studies ranged from lipid bilayers and liposomes to both pro- and eukaryotic cells in culture and various tissues *in vivo*. Multiple theoretical studies explored the phenomenon of electropermeabilization by molecular dynamics, sophisticated electrical circuit modeling, and numerical simulations. Still, the mechanisms of electroporation itself and of electroporation-induced biological phenomena have not been fully understood, which stimulated a new surge of interest in the topic and numerous recent publications, e.g. [1]–[16].

Notwithstanding gaps in knowledge, electroporation has both well-established and developing applications for gene electrotransfer and gene therapy [14], [16]–[19] cell fusion [20]–[22], electrochemotherapy [23], [24], tumor ablation [3], [5], [13], [25], vascular smooth muscle cells ablation [4], sterilization [26], [27], and food processing [28], [29]. Numerous studies have focused on optimization of exposure conditions to produce maximum desired effect while minimizing side effects. However, with multiple parameters to consider (E-field, pulse duration, number of pulses, their shape and repetition frequency) these studies have been laborious and showed limited success. The optimization process remains mostly empirical, whereas quantitative and mechanistic principles that determine the outcome of EP exposures are debated [7]–[10], [15], [30]–[32].

Out of different EP exposure parameters, the impact of the pulse repetition frequency (PRF) is the least understood, resulting in controversial findings and treatment recommendations. Aside from the trivial heating effect that increases with increased PRF (less time for heat dissipation), experimental and theoretical studies using different endpoints reported significantly greater bioeffects at higher PRF [2], [13], [16], [30], [33]–[35], significantly greater effects at lower PRF [1], [4], [5], [10], [25], [29], [36]–[38], biphasic or more complex dependences [30], [38], [39], or relatively little role of PRF within studied limits [2], [8], [38], [40].

Specifically, Vernier et al. [34] reported significant uptake of membrane impermeable dyes (propidium and YO-PRO-1) when Jurkat cells were exposed to 30 pulses (4-ns duration, 80 kV/cm) at 1 and 10 kHz rates. No dye influx was detected at the lower rates of 10 and 100 Hz. Jiang and Cooper [35] showed the reduction of the E-field threshold for nociceptor excitation from 30 to 24 and to 16 V/cm as the PRF was increased from 100 Hz to 1 and 4 kHz, respectively (for a train of one hundred 12-ns pulses). Similarly, applying 100-µs pulses at intervals under 1 ms lowered the electroporation threshold of artificial bilayer lipid membranes [33].

Increasing PRF from 0.1 to 1, 10, and 77 Hz (six 1-ms pulses at 800 V/cm) decreased the 24-hr survival of exposed CHO cells from 60% (0.1 Hz) to about 20% (77 Hz) [16]. Likewise, a train of 2,000 pulses (100-ns duration, 30 kV/cm) was more efficient in eliminating murine melanomas at PRF of 5 and 7 Hz when compared to 1 or 3 Hz [13]; however, the statistical significance of this finding was not evaluated. Overall, higher efficiency of higher PRF is usually attributed to the temporal summation of brief subthreshold effects (or lesions) which can recover without consequences if the interval between pulses is sufficiently long.

In contrast to the above studies, Rubinsky and co-authors observed more efficient cell killing at lower PRF, both *in vitro* and *in vivo* [4], [5], [25], [37]. The authors typically adjusted several exposure parameters at once (in order to keep the cumulative EP duration or the total dose unchanged), so isolating “pure” effects of PRF may be not straightforward. Still, one can find that, for example, eight 1-ms pulses at 2.5 kV/cm were more efficient at 0.03 Hz than at 0.3 Hz; or eighty 100-µs pulses at 2.5 kV/cm were more efficient at 0.3 Hz than at 3 Hz (see Fig. 2 in [25]). When the delivered energy was kept constant, longer exposures at the lower E-field and using greater number of pulses typically were more efficient, despite lower temperature rise. Gradual enhancement of the cytotoxic effect as the PRF decreased from 5 kHz to 1 kHz, 60 Hz, and 1 Hz was also reported in SKOV3 cells exposed to exponentially-decaying EPs [36]. The reason for higher efficiency of lower PRF has not been identified, but it may be related to the reduction of EP efficiency when the cell membrane is made “leaky” by the previous EPs [9], [39]. With longer inter-pulse intervals, the membrane partially reseals and the efficiency of the coming pulses increases. Simulation studies showed an overall slow decrease of the fractional area of pores with PRF increase, however, with sharp regular troughs at certain frequencies [39].

Pucihar and co-authors [38] found that the uptake of Lucifer Yellow dye by DC3F cells exposed to 26 pulses of 30-µs duration was the same for 1 Hz and 8.3 kHz, however, it required about 1.5 times higher E-field for the higher PRF. The dependence was similar for 100-µs pulses, except for a slightly higher dye uptake at 10 Hz compared to both lower (1 Hz) and higher PRF (1 and 2.5 kHz).

For a train of 200 pulses of 50-µs at 0.9 kV/cm, the cytotoxic effect in CHO cells showed a bell-shaped dependence on PRF: it was weaker at the central frequency of 10 Hz, and gradually enhanced as the rate either decreased to 0.5 Hz or increased to 100 Hz [30]. At the same time, propidium uptake by the cells was flat for the range from 0.5 to 20 Hz, and increased at 50 and 100 Hz. The authors hypothesized that the increased cytotoxicity at the lowest PRF may be related to slow rotation of cells in suspension, so that different portions of their membrane get exposed to the field and more membrane is porated. This idea was later extended into a complex model that related random statistical rotations of suspension cells to EP efficiency [10].

In the field of electrochemotherapy, a significant effort has been made in recent years to compare 1 Hz and 5 kHz delivery rates of 100-µs pulses. The advantage of the 5 kHz PRF was alleviation of pain and discomfort from EP application, whereas its anti-tumor efficiency was either similar, or somewhat higher, or somewhat lower, depending on the concurrent conditions and the method of assessment (for discussion, see [1], [40]–[42]).

Importantly, none of the above-mentioned studies was specifically focused on the role of PRF; instead, it was just one of variables evaluated alongside other parameters of interest. The lack of comprehensive, wide scale studies may explain, at least partially, the controversy concerning PRF impact and mechanisms involved. The present study was originally conceived just to fill this void; unexpectedly, it revealed an all-new phenomenon of EP-induced sensitization to subsequent EP deliveries. This gradually developing sensitization is a biological response of living cells and, not surprisingly, it was not predicted or considered by any models which viewed EP effects solely as an electrochemical process. Taking sensitization into account greatly complements understanding of the reported electroporation findings and helps to design most effective EP treatment regimens for various applications.

## Results

### Effect of PRF on long-term cell survival

**Figure 1:**
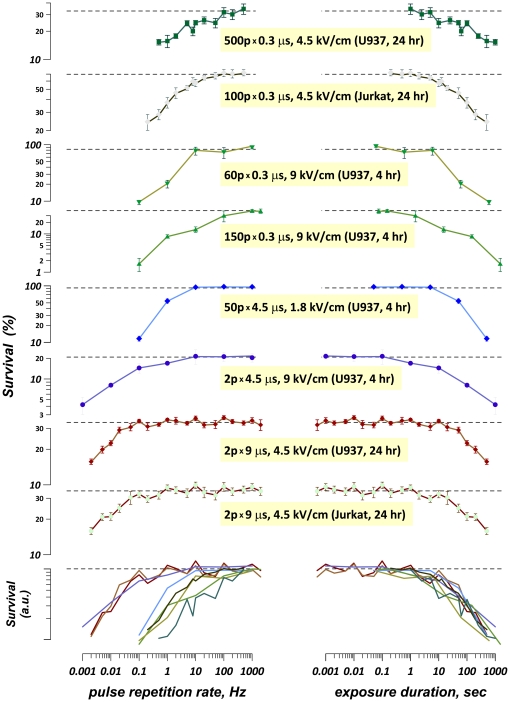
The effect of the pulse repetition rate (left column) and of the total duration of the treatment (right column) on cell survival. Each plot (except those at the bottom) represents a separate series of experiments where cells were exposed to a fixed number of pulses of a given amplitude and duration (see legends within the figure; e.g., for the top plot the legend means “500 pulses of 0.3 µs duration at 4.5 kV/cm). The only variable in each series was the pulse repetition rate and, consequentially, the total duration of the treatment. Other data in the legends are the cell type and the timepoint after exposure when the cell survival was measured. At 4 hr, cell survival was measured using dye exclusion/quenching method; at 24 hr, it was measured using MTT assay (see “Methods” for detail). Each datapoint is the mean +/− s.e for 3–12 independent experiments. For the bottom plots, the curves from all series of experiments were collapsed together; shown are only the connecting lines; the mean value symbols and error bars have been omitted for clarity. See text for more detail.

Fig. 1 (left column) summarizes the results of multiple experiments that tested the cytotoxic efficiency of EP trains delivered at different PRF. Each plot corresponds to an independent set of experiments where the E-field, pulse duration, and the number of pulses were kept constant, while the PRF was the only parameter varied. Overall, these experiments explored rather diverse exposure conditions: 300 ns to 9 µs pulse duration, 1.8 to 9 kV/cm E-field, 2 to 500 pulses per train, at 0.001 to 1,000 Hz PRF. In addition, the experiments were performed in two cell lines (Jurkat and U937), and cell survival was measured by different methods.

Regardless of the specific conditions tested, maximum cell killing in all experiments was achieved at the lowest tested PRF. As the PRF increased, the effect gradually weakened and reached a plateau at higher frequencies. Surprisingly, the level at which the plateau was reached differed more than 1000-fold from one set of experiments to another. The bottom graph in the left column of Fig. 1 shows that the increase of the EP efficiency from the minimum plateau level occurred at PRFs as different as 0.1–500 Hz. Such diversity was difficult to explain, and it also suggested that perhaps it was not the PRF that actually determined the increased effect at the lower pulse rates.

To check this idea, the same data were re-plotted against the total duration of the treatment, which was simply a ratio of the pulse number to the PRF (Fig. 1, right column). The data from the different sets of experiments now showed much better agreement: in all cases, the transition from the plateau to higher cytotoxic efficiency corresponded to the treatment duration of about 10 sec (Fig. 1, bottom graph in the right column).

Interpretation of this finding is easier when considering the exposures that consisted of only two pulses (three graphs immediately above the combined plots). In this case, the treatment duration was simply the interval between the pulses, and the cytotoxic efficiency increased once a certain interval was exceeded. These data prompt that the first pulse somehow conditioned the cells, making them more sensitive to the second pulse if it is delivered after a proper delay. The validity of this explanation also for multi-pulse treatments is confirmed below by exposure fractionation.

### Fractionated exposure: a new approach to increase the EP efficiency

**Figure 2:**
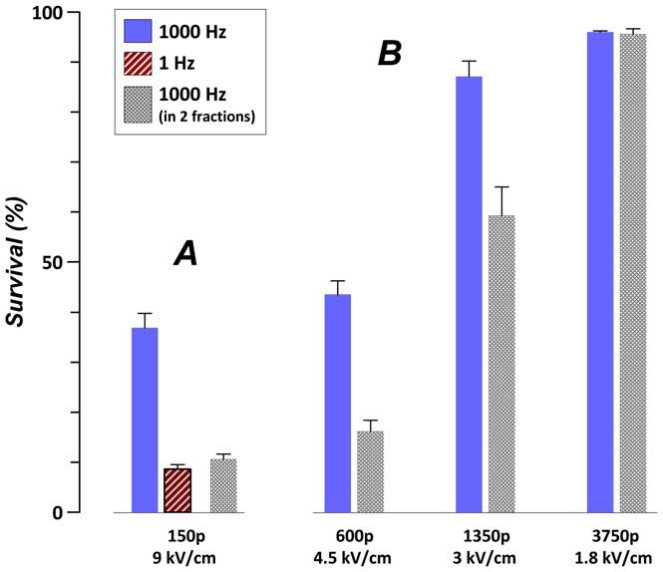
Enhancement of the EP cytotoxic effect by exposure fractionation. U937 cells were exposed 0.3-µs EPs; the pulse number, amplitude, and delivery mode, are indicated in the figure. Cell survival was measured as a percentage of propidium-excluding cells at 4 hr post exposure (mean +/− s.e., n = 3–7). Survival in sham-exposed samples was over 95% (data not shown). ***A***: Exposure to 150 pulses was significantly more effective at 1 Hz than at 1,000 Hz. However, splitting the 1,000-Hz train in two fractions of 75 pulses each, with a 150-s interval, made it as efficient as the 1-Hz treatment. ***B***: Splitting of a single high-rate train in two same size fractions with 150-s interval enhanced the effect EP of 4.5 and 3 kV/cm EPs, but not at the lower EP amplitude of 1.8 kV/cm.

We showed in Fig. 1 that the EP cytotoxic effect is enhanced when the total duration of the treatment exceeds a certain minimum. To test if this enhancement was indeed unrelated to a change in PRF, we needed to increase the treatment duration while keeping both the PRF and the number of pulses constant. The only feasible way to do it was splitting of a high rate EP train into separate fractions separated by a long quiescent period. The effect of such exposure fractionation is illustrated in Fig. 2, A. The same train of 150 pulses (300 ns, 9 kV/cm) was far more efficient at 1 Hz than at 1,000 Hz; the respective train durations and cell survival levels were 150 s and 8.4+/−1.1% versus 0.15 s and 37+/−2.8% (p<0.01, Student's t-test). However, when the 1,000-Hz train was split into two fractions (75 pulses, 0.75 s each) separated by a 150-s interval, the resulting cell survival dropped to 10.6+/−1.1%, i.e., it became the same as after the 1-Hz, 150-s exposure.

This finding unequivocally demonstrates that it was indeed the treatment duration, rather than a particular PRF, that determined the enhancement of the cytotoxic effect. When the duration of the 1,000-Hz EP treatment was increased by splitting one train in two fractions to match the duration of the 1-Hz treatment, both the 1000-Hz and 1-Hz exposures had the same effect.

Fig. 2, B shows that fractionation enhanced the effect for different exposure conditions, but excluding those when the E-field was reduced to a sub-threshold value (1.8 kV/cm). When a single train had no appreciable effect on cell survival, splitting it in fractions had no additional effect.

However, the lack of appreciable cytotoxic effect of a high-PRF exposure does not necessarily always indicate the lack of subthreshold lesions. Under certain conditions, such subthreshold lesions can be amplified by slower or fractionated treatments to cause a profound drop in survival (e.g., see Fig. 1, 5th curve from the top).

**Figure 3:**
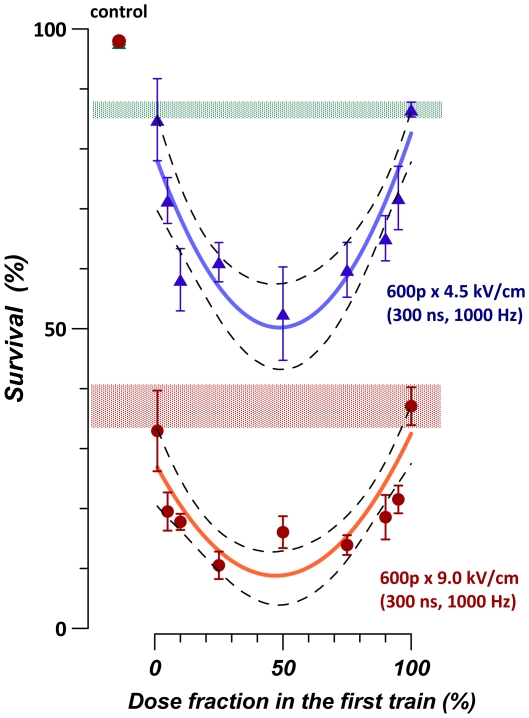
The role of fraction size in the enhancement of the EP effect by fractionation. Survival of U937 cells was determined by propidium exclusion at 4 hr following exposure to 0.3 µs EPs at either 4.5 kV/cm (top graph) or 9 kV/cm. The total number of 600 pulses was split into two fractions which were delivered with a 6-min interval. The number of pulses in the first train varied form 1% to 100% of the total. The latter value corresponded to delivering all pulses in a single train, and the respective survival levels are shown by shaded areas. Mean+/− s.e., n = 4–6. Solid lines are best fit approximations using second degree polynomial function. Dashed lines delimit the borders of 95% confidence intervals for the best fit.

We further demonstrated that the enhancement of EP effect by exposure fractionation depends on the fraction size (Fig. 3). A train of 600 pulses (300 ns, 4.5 or 9 kV/cm) was delivered either as a single train (100% pulses in the first train), or as two trains of varied pulse contents, with a 6-min interval between the trains. For example, 1% dose fraction in the 1st train corresponded to 6 pulses in the first train followed by 594 in the 2nd train 6 min later; 10% was 60 pulses in the 1st train and 540 in the 2nd one, and so forth. Although the best fit curves showed maximum efficiency as dose fraction ratio approached 50%, the effect was essentially flat within a wide range of dose fractions, from 10–20 to 80–90%. However, with the smallest fraction (1%), the effect was not different from a single-train exposure, irrespective of the fact that the fractionated exposure lasted 6 min and the single train was delivered in 0.6 s.

### EP-induced propidium uptake and membrane rupture in substrate-attached cells

Several earlier studies attributed the enhanced effect of low PRF to slow, random rotation of cells in suspension, so that different portions of cell membrane face the electrodes and get permeabilized by EP [10], [30]. While this explanation did not explain well our observations, the only way to unequivocally rule out the impact of rotation was to replicate the findings in substrate-attached cells. In addition, it was deemed important to replicate the principal findings about PRF and fractionation using a different EP generation and delivery setup, different experiment protocol, and making measurements in individual selected cells rather than in bulk suspension.

**Figure 4:**
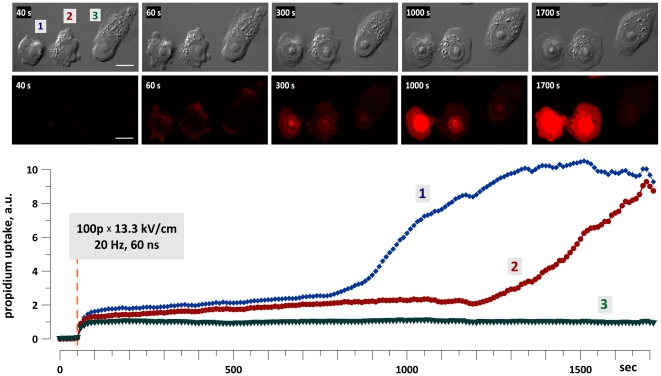
Two distinct modes of propidium uptake in EP-exposed cells. A group of three CHO cells attached to a coverslip was exposed to an EP train at 50 s into the experiment. Exposure parameters are indicated in the figure. Insets show differential-interference contrast and fluorescent images of cells at selected timepoints. Scale bars: 10 µm. The images were taken every 10 s throughout the experiment. The graph shows the time dynamics of Pr uptake by cells 1, 2 and 3. Note the immediate increase in Pr fluorescence in all three cells, caused by electropore opening and transient Pr uptake. Following the transient uptake, the intensity of fluorescence displayed little changes until membrane rupture and massive Pr entry in cells 1 and 2, but not in cell 3.

We used a confocal microscope setup for EP exposure of individual cells to monitor and quantify propidium (Pr) uptake in CHO cells attached to a coverslip. At high enough treatment intensity (we used a train of 100 pulses, 60 ns duration, at 13.3 kV/cm), the exposure could cause two distinct types of Pr uptake: a transient uptake (during and immediately after EP) and a delayed, accelerated uptake (Fig. 4).

The transient uptake was characterized by an abrupt increase of the fluorescent signal during and immediately after the exposure, reflecting Pr entry and binding to nucleic acids. Pr fluorescence due to the transient uptake reached a plateau within about 1 min, and remained at this level for the rest of the observation period (30 min).

In some EP-treated cells, the transient uptake was eventually followed by a delayed, more intense, and gradually accelerating Pr uptake (until reaching the detector saturation). Whereas the transient uptake is an immediate and direct manifestation of electroporation, the delayed/accelerated uptake is a sign of irreversible membrane rupture when a cell fails to promptly repair the EP-induced membrane lesions. The development of the delayed/accelerated Pr uptake was probably a result of multiple parameters (such as cell shape, size, exact position with respect to the electrodes, stage in cell cycle, shielding by other cells), but showed no clear association with any of them. These two modes of Pr uptake were also reported by other investigators for nano- and microsecond duration EP treatments [43]; the most likely reason for delayed membrane rupture is limited electropore permeability and gradual cell swelling by the colloid osmotic mechanism [44], [45].

**Figure 5:**
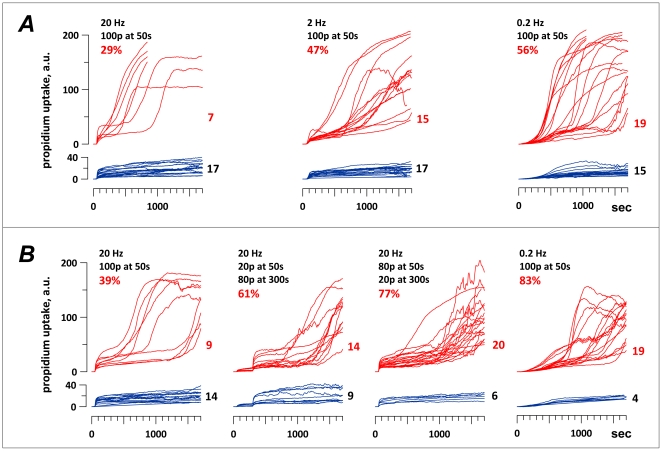
The effect of PRF (A, B) and exposure fractionation (B) on the incidence of delayed membrane rupture in EP-exposed CHO cells. ***A*** and ***B*** are two separate and independent series of experiments; within each series, different exposure regimens were alternated in random order. Membrane integrity was probed by Pr uptake; each curve corresponds to Pr uptake in an individual exposed cell, as measured by cell imaging every 10 s throughout the experiment. EP exposure caused transient Pr uptake due to electroporation in all cells and delayed/accelerated Pr uptake due to secondary membrane rupture in some cells (see Fig. 4 and text for more detail). For clarity, for each type of treatment, cells that showed only transient Pr uptake (blue curves, bottom graphs) are separated from cells that showed both transient and delayed Pr uptake (red curves, top graphs). The number of cells that fell into each of the two categories is shown to the right of the graphs. In all groups, cells were exposed to 100, 60-ns pulses at 13.3 kV/cm, whereas the PRF and pulse delivery protocols varied (see legends in the figure). The legends also give the percent of ruptured cells in each group. Note the increase of EP exposure efficiency at lower PRF (***A*** and ***B***) and also when the 20-Hz train was split in two fractions (***B***). Single-train 20- and 0.2-Hz exposures in ***A*** and ***B*** are identical treatments, although in ***B*** both showed somewhat higher efficiency.

Fig. 5 compares the incidence of membrane rupture (as reflected by the delayed Pr uptake) for different rates and protocols of EP exposure, when the E-field, pulse duration, and pulse number were kept the same. For better viewing, cells that showed only transient uptake are separated from those that developed both transient and delayed uptake.

Fig 5, ***A*** shows that the incidence of membrane rupture resulting from exposure to a single EP train (100 pulses at 13.3 kV/cm, 60-ns pulse duration) increased with decreasing the PRF, namely from 29% at 20 Hz to 47% at 2 Hz, and 56% at 0.01 Hz. Same as in the cell survival experiments described above, the lowest PRF was the most efficient.

Fig 5, ***B*** shows a separate set of experiments, where 20 Hz and 0.2 Hz treatments served both as an independent replication of the experiments in Fig. 5, ***A***, and as reference points for efficiency of the fractionated 20-Hz exposures. For unknown reasons, the effects of both 20- and 0.2-Hz exposures in set ***B*** were somewhat greater than previously in ***A*** (p>0.05), but the higher efficiency of the 0.2 Hz compared to 20 Hz (single train) remained very consistent (p<0.002 for A and B data pooled together, two-tailed Fisher Exact Probability test). Both fractionated 20-Hz exposures (20+80 pulses or 80+20 pulses, with 250-s interval) were more effective than a single 20-Hz train, approaching the efficiency of the 0.2-Hz exposure.

Overall, the PRF and dose fractionation had the same effect in the attached cells as in the suspended cells (as described in the previous sections), despite looking at a different endpoints, using shorter (60-ns) EP, and employing an entirely different setup for EP generation and delivery.

### Effective dose reduction by fractionation of exposure

In experiments described above, we changed pulse delivery protocols while keeping the exposure dose constant (including same E-field, same number and duration of pulses). Now, we chose two exposure protocols (a single high-rate train versus same train split in two fractions) and compared their efficiencies within a wide range of doses.

**Figure 6:**
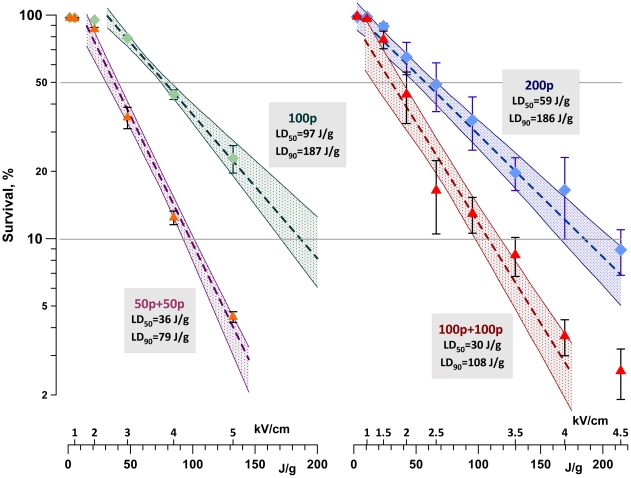
Effective reduction of the lethal dose (LD) by exposure fractionation. U937 cells were exposed to 100 (left) or 200 (right) of 0.3-µs duration EPs. The exposure was delivered either as a single train or as two equal fractions (50+50 and 100+100) with a 5-min interval. EPs were applied at different E-fields amplitudes (values are given above the abscissa), resulting in different absorbed doses. The graphs show cell survival (mean+/− s.e., n = 3–6) versus the dose for different EP treatments. Dashed lines are the best fit data approximations using exponential function; shaded areas denote 95% confidence intervals. Cell survival was measured by propidium exclusion at 4 hr post exposure. Legends show LD values for elimination of 50% and 90% of cells (LD_50_ and LD_90_) by the tested exposure protocols.

As shown in Fig. 6, the same cytotoxic effect was achieved at significantly lower doses when using the fractionated treatment. Doses that killed 50% and 90% of cells (LD_50_ and LD_90_) were 2–2.5 times lower for the fractionated exposures. This result can potentially be further improved by careful optimization of different exposure parameters; at this time, the 2-fold dose reduction can serve as a ballpark estimate of the benefits that can be achieved by exposure fractionation in various EP applications.

## Discussion

The principal finding of our study is that the efficiency of varied EP treatments increased when the treatment duration was made sufficiently long, either by applying pulses at a low rate, or by splitting a single pulse train into separate fractions. This increase in efficiency is explained by a gradual and delayed increase in the EP susceptibility during the exposure procedure. In other words, the EP (or EPs) that are delivered first cause sensitization to subsequently delivered EPs, thereby increasing their effect and making the entire EP treatment more efficient. It is well established that the principal and primary effect of intense electric pulses in living cells is electroporation of the cell plasma membrane, which allows us to interpret the observed increase in EP efficiency as the electroporation-induced electrosensitization.

In the Introduction, we have discussed the studies that attempted to explain various pulse rate effects relying solely on the physical effects of the applied electric field. However, none of these explanations can explain the delayed sensitization (which requires at least 10–100 sec of the total treatment duration) or predict the effect of the fractionated treatments. The data point to a biological rather than merely physical mechanism of sensitization, and below we discuss several hypothetic scenarios of what cellular events could lead to sensitization.

Once the cell is electroporated by the first pulse(s) of the EP exposure, the ions from the extracellular medium diffuse into the cell, down the concentration and electrochemical gradients, whereas the intracellular ions leave the cell. The cell attempts to restore the ion gradients and membrane potential by activating ion pumps and repairing the membrane [46], [47]. High energy expenditure for these processes may be aggravated by ATP leakage out of cell through the electropores. One can speculate that a prolonged high demand for ATP combined with ATP loss during longer exposures could be a factor responsible for sensitization.

Among ions that can enter through the electropores, Ca^2+^ will have multiple effects on cell physiology. It is not clear how exactly the increased Ca^2+^ concentration would change the cell susceptibility to EP, but it should come as no surprise that prolonged time intervals when the internal Ca^2+^ is elevated (e.g., due to a longer EP treatment) may be unfavorable for cells and make them more vulnerable.

Plasma membrane permeabilization also triggers cell volume changes due to the so-called colloid osmotic mechanism [45], [48]. In a “typical” bath buffer, as well as in the cell growth medium, permeabilization leads to water uptake and cell swelling. The increase of cell diameter translates into a higher induced membrane potential when next EPs are applied [22], which makes electroporation more efficient. Furthermore, the additional membrane for cell swelling is recruited from cytoplasmic invaginations of the plasma membrane, and this “spreading out” of the membrane could also contribute to increasing EP effects.

Notably, considering these mechanisms helps to reconcile the seemingly contradictory studies that reported diverse effects of PRF. For example, Faurie and co-authors [16] reported greater cytotoxic effect of 1-ms pulses as the PRF was increased from 0.1 to 77 Hz, which is the opposite of what was shown in our work and other studies [4], [5], [25], [37]. It appears that the critical distinction of this study was composition of the pulsing medium, which contained 250 mM sucrose, 1 mM MgCl_2_, and 10 mM of K_2_HPO_4_/KH_2_PO_4_ buffer [16]. In this medium, membrane electroporation obviously will not lead to any Ca^2+^ or Na^+^ uptake, and cell swelling will be weaker or even replaced by shrinking. Thus, the lack of electroporation-induced electrosensitization in this pulsing medium is consistent with the involvement of one or several mechanisms mentioned above.

Another potential mechanism of electrosensitization may involve direct or indirect oxidative damage to membrane by EP exposure [49]–[51], which would enhance its susceptibility to permeabilization by EPs [52].

Although the exact sequence of events resulting in electrosensitization has yet to be identified, taking this phenomenon into account helps to explain contradictions in published data, and will likely be beneficial for many existing and coming applications of electroporation. Controlled cell destruction with minimum side effects and energy expenditure is the principal endpoint in tissue and tumor ablation, sterilization, and food processing, whereas in other applications such as gene electrotransfer, the cell death is a major outcome to avoid. Engaging (or preventing) electrosensitization by changing pulse rate and by exposure fractionation can be a simple and efficient approach to achieve the desired goals of various electroporation treatments.

## Materials and Methods

### Cell lines and propagation

Experiments were performed in two suspension cell lines (Jurkat clone E6-1, human T-lymphocytes, and U-937, human monocytes) and one anchor-dependent cell line (CHO-K1, Chinese hamster ovary). The cells were obtained from ATCC (Manassas, VA) and propagated at 37°C with 5% CO_2_ in air according to supplier's recommendations. Jurkat and U-937 cells were grown in RPMI-1640 medium supplemented with 10% fetal bovine serum and 2 mM L-glutamine. CHO cells were propagated in Ham's F12K medium supplemented with 10% FBS. The media also contained 1% penicillin/streptomycin. The media and its components were purchased from Mediatech Cellgro (Herdon, VA) except for serum (Atlanta Biologicals, Norcross, GA).

### EP exposure and viability assays for suspension cell lines

Cells were harvested during the logarithmic growth phase, pelleted by centrifugation, and resuspended in fresh growth medium at either 0.6 or 1.2×10^6^ cells/ml. The suspension was dispensed into conventional electroporation cuvettes with 1- or 2-mm gap between the electrodes (BioSmith Biotech, San Diego, CA). The cuvettes were exposed to EPs at room temperature (21–23°C), one cuvette at a time. The exposure protocols were organized so that to (1) minimize waiting of aliquoted cells for EP exposure to less than 10 min (on a few occasions, up to 20 min), (2) ensure the same treatment conditions for parallel samples, so that the only variable would be the EP exposure regimen, and (3) carefully randomize all EP treatments, including “sham” exposures.

Unipolar EPs of 300-ns, 4.5- or 9.0-µs duration and up to 1-kV amplitude were generated by an AVTECH AVOZ-D2-B-ODA pulser (AVTECH Electrosystems, Ottawa, Ontario, Canada). To produce pulse trains of predetermined duration at selected pulse repetition rates, this generator was triggered externally from a model S8800 stimulator (Grass Instruments Co., Quincy, MA). The pulse amplitude and shape (trapezoidal, with rise and fall times (20%–80%) of <100 ns) were monitored using a Tektronix TDS 3052B oscilloscope. Pulses were delivered to the electroporation cuvette using a 50- to 10-Ohm transition module (AVOZ-D2-T, AVTECH Electrosystems) modified into a cuvette holder. The E-field values were obtained by dividing the mean pulse voltage (as measured by the oscilloscope) by the width of the gap in the electroporation cuvette. The absorbed dose was calculated as the energy delivered to the sample normalized to the mass of the sample [49].

Sample temperature during and after EP exposure was checked with a fiber optic ReFlex-4 thermometer (Nortech Fibronic, Quebec City, Quebec, Canada), and never exceeded 30°C.

Cell survival was measured either in 24 hr post exposure using MTT (3-(4,5-Dimethylthiazol-2-yl)-2,5-diphenyltetrazolium bromide) assay, or in 4 hr post exposure by a fluorescent dye exclusion/quenching method.

For the MTT assay (BioAssay Systems, Hayward, CA), exposed cells were aseptically aliquoted into a 96-well plate, in triplicates at 50×10^3^ cells/well, and diluted to 100 µl with fresh growth medium. The plate was incubated at 37°C, with 5% CO_2_ in air. At 20 hr after EP treatment, 10 µl of the MTT reagent were added to each well, and incubation continued for additional 4 hr. Formed blue formazan crystals were dissolved by adding the solubilization buffer (100 µl/well) and placing the plate on an orbital shaker overnight. Absorbance at 570 nm was read the next day using Synergy 2 microplate reader (BioTEK, Winooski, VT).

With the MTT assay, cell survival was considered proportional to the sample absorbance, and was expressed in % to the absorbance of the sham-exposed parallel control samples. The results are presented in the graphs and text as mean values +/−s.e. for a minimum of three independent experiments (usually 5–12 experiments).

For the dye exclusion/quenching method, aliquots of exposed cells were transferred into microcentrifuge tubes and left in the incubator until analysis (the tube lid was left open). At 4 hr post exposure, 20 µl of the cell suspension were mixed with equal volume of staining solution (100 µg/ml of propidium iodide and 0.5 µg/ml of acridine orange in phosphate-buffered saline). The dyes and chemicals were purchased from Sigma–Aldrich (St. Louis, MO). The sample was immediately loaded into a counting chamber of the automated cell counter Cellometer Vision with two-channel cell fluorescence detection (Nexcelom Bioscience LLC, Lawrence, MA).

Both employed dyes markedly increase fluorescence upon binding to DNA; while acridine orange readily penetrates the intact cell membrane, propidium does not. Live cells were distinguished by bright fluorescence of acridine orange (excitation/emission 475/535 nm). In cells with damaged membrane (presumably dead), this signal was quenched by fluorescence resonance energy transfer to propidium that entered the cell and bound to DNA. Combined fluorescence of either acridine orange or propidium (excitation/emission 525/595 nm) was used to determine the total (live + dead) cell count.

When using the dye exclusion/quenching assay, cell survival was expressed as a percentage of live cells to the total number of cells counted in each slide (usually several hundreds of cells), and two slides were processed for each datapoint. The survival data were presented as a mean+/− s.e for 3–7 independent experiments.

### EP exposure of individual cells on a coverslip and fluorescent microscopy

The procedures were similar to those described recently [11], [53]. For the passage immediately preceding experiments, CHO cells were transferred onto glass cover slips pre-treated with poly-L-lysine to improve cell adhesion. After several hours, a cover slip with cells was transferred into a glass-bottomed chamber (Warner Instruments, Hamden, CT) mounted on an Olympus IX71 inverted microscope equipped with an FV 300 confocal laser scanning system (Olympus America, Center Valley, PA). The chamber was filled with a buffer composed of (in mM): 136 NaCl, 5 KCl, 2 MgCl_2_, 2 CaCl_2_, 10 HEPES, and 10 Glucose (pH 7.4), with addition of 30 µg/ml of propidium iodide. The buffer osmolality was at 290–300 mOsm/kg, as measured with a freezing point microosmometer (Advanced Instruments, Inc., Norwood, MA). The chemicals were purchased from Sigma–Aldrich.

EPs were delivered to a selected cell (or a group of 2–4 cells) with a pair of tungsten rod electrodes (0.08-mm diameter, 0.15 mm gap). With a help of a robotic micromanipulator (MP-225, Sutter, Novato, CA), these electrodes were positioned precisely at 50 µm above the coverslip surface so that the selected cells were in the middle of the gap between their tips. Nearly rectangular 60-ns pulses were generated in a transmission line-type circuit, by closing a MOSFET switch upon a timed delivery of a TTL trigger pulse from pClamp software via a Digidata 1322A output (MDS, Foster City, CA). The exact PRF, the EP delivery protocol, and synchronization of EP delivery with image acquisitions were programmed in pClamp.

The E-field between the electrodes was determined by 3D simulations with a finite element Maxwell equations solver Amaze 3D (Field Precision, Albuquerque, NM). The exact EP shapes and amplitudes were captured and measured with a Tektronix TDS 3052 oscilloscope.

Differential-interference contrast and fluorescent images of cells (excitation: 488 nm; emission 605 nm) were collected every 10 sec (starting exactly 50 s prior to the first EP) using a 60x, 1.42 NA oil objective. Photomultiplier tube settings were biased towards high sensitivity and detection of even minimal propidium uptake, although massive uptake caused detector saturation. Images were quantified with MetaMorph v. 7.5 (MDS). All experiments were performed at a room temperature of 22–24°C.
